# A Novel Approach for Multiple Material Extrusion in Arthroscopic Knee Surgery

**DOI:** 10.1007/s10439-022-03061-5

**Published:** 2022-09-03

**Authors:** Tommaso Mazzocchi, Daniele Guarnera, Diego Trucco, Francesco Rocco Restaino, Lorenzo Vannozzi, Alessio Siliberto, Gina Lisignoli, Stefano Zaffagnini, Alessandro Russo, Leonardo Ricotti

**Affiliations:** 1 The BioRobotics Institute, Scuola Superiore Sant’Anna, Piazza Martiri della Liberta’ 33, 56127 Pisa, Italy; 2 Department of Excellence in Robotics & AI, Scuola Superiore Sant’Anna, Piazza Martiri della Liberta’ 33, 56127 Pisa, Italy; 3 IRCCS Istituto Ortopedico Rizzoli, SC Laboratorio di Immunoreumatologia e Rigenerazione Tissutale, Via di Barbiano, 1/10, 40136 Bologna, Italy; 4 IRCCS Istituto Ortopedico Rizzoli, Orthopaedic and Traumatologic Clinic, Via di Barbiano, 1/10, 40136 Bologna, Italy

**Keywords:** Cartilage regeneration, Biomaterials, Arthroscopic tools, Arthroscopic surgery, Osteoarthritis, Medical devices

## Abstract

Articular cartilage defects and degenerative diseases are pathological conditions that cause pain and the progressive loss of joint functionalities. The most severe cases are treated through partial or complete joint replacement with prostheses, even if the interest in cartilage regeneration and re-growth methods is steadily increasing. These methods consist of the targeted deposition of biomaterials. Only a few tools have been developed so far for performing these procedures in a minimally invasive way. This work presents an innovative device for the direct deposition of multiple biomaterials in an arthroscopic scenario. The tool is easily handleable and allows the extrusion of three different materials simultaneously. It is also equipped with a flexible tip to reach remote areas of the damaged cartilage. Three channels are arranged coaxially and a spring-based dip-coating approach allows the fabrication and assembly of a bendable polymeric tip. Experimental tests were performed to characterize the tip, showing the ability to bend it up to 90° (using a force of ~ 1.5 N) and to extrude three coaxial biomaterials at the same time with both tip straight and tip fully bent. Rheometric analysis and fluid-dynamic computational simulations were performed to analyze the fluids’ behavior; the maximum shear stresses were observed in correspondence to the distal tip and the channel convergence chamber, but with values up to ~ 1.2 kPa, compatible with a safe extrusion of biomaterials, even laden with cells. The cells viability was assessed after the extrusion with Live/Dead assay, confirming the safety of the extrusion procedures. Finally, the tool was tested arthroscopically in a cadaveric knee, demonstrating its ability to deliver the biomaterial in different areas, even ones that are typically hard-to-reach with traditional tools.

**Supplementary Information:**

The online version contains supplementary material available at 10.1007/s10439-022-03061-5.

## Introduction

Articular cartilage defects and degenerative joint lesions are widely spread pathological conditions.^18^ They cause pain and loss of joint functionality, also constituting a factor for the onset of chronic diseases such as osteoarthritis (OA). The quality of life is severely affected by this issue, especially in elderly adults: almost 10% of men and 18% of women over 60 suffer the effects of acute cartilage damages.^20^ Widuchowsky et al. found chondral lesions in 60% of a large number of knee arthroscopies (25,124), and the average age of these patients was 39.^52^

Depending on the severity of the lesion,^30,45,48^ different solutions have been proposed as a treatment.^5,19,53^ For high severity levels, joint replacement with prostheses represents the most common procedure to re-establish the correct knee functionality.^8^ However, the duration of the prosthesis is limited and presents infection risks.^53^ Another relevant drawback of these procedures is the need to be performed in open surgery. They require, in fact, a long surgical intervention time, thus resulting extremely invasive and traumatic for the patient.^24,54^

During the last decades, tissue engineering has focused on new methods for the re-growth of healthy cartilage tissues, fostering its regeneration.^2,23^ The targeted deposition of both cell-laden and cell-free biomaterials endowed with regenerative cues has become a concrete option to pursue.^47^ Several efforts have been made in recent years to develop novel materials (e.g., based on hyaluronic acid) aiming at regenerating the cartilage tissue, but often based on ex situ fabrication techniques.^1^ At present, only a few strategies and tools have been developed for a reliable deposition of biomaterials and cells onto the cartilage lesions so far. O’Connell, Duchi, Di Bella, and colleagues recently proposed devices for in situ bioprinting on the cartilage,^15,16,38^ featured by two coaxial conduits to protect the central channel (which is aimed to transport the cells) through an outer shell layer of a different biomaterial. This external layer would act as a protective layer for the internal cell-laden biomaterial, minimizing the shear stresses on the cells during the delivery. It has been argued that, during extrusion, another hydrogel may also be used to guarantee that the cell-laden biomaterial delivered will remain attached to the injury site thanks to the adhesive properties of the layer used as a primer.^47^

While these approaches are exciting, they still typically require an open-surgery approach. Therefore, an evolution of this concept toward an arthroscopic scenario would be highly desirable.

In general, the arthroscopic procedures need to face many challenges, such as the inaccessibility of far joint areas.^3^ Desirable properties of the tool are flexibility, lightweight and wide range of motion, which are essential for its implementation .^56^ Arthroscopic surgical tools having orientable tips have been recently developed.^9,39,40,42^ However, none of them is designed for delivering biomaterials. The possibility to deliver the targeted biomaterial arthroscopically even in hard-to-reach areas would indeed imply higher effectiveness of the treatment. Thus, in this framework, the combination of intelligent material deposition and high maneuverability of the device in an arthroscopic scenario is a challenge still not accomplished.

This work presents an innovative strategy for minimally invasive treatment of cartilage lesions through a smart arthroscopic extruder. This device can deliver up to three materials simultaneously in a concentrical arrangement, and it is equipped with a flexible and orientable tip to reach hardly accessible regions of the joint.

## Materials and Methods

### Concept Description

**Figure 1:**
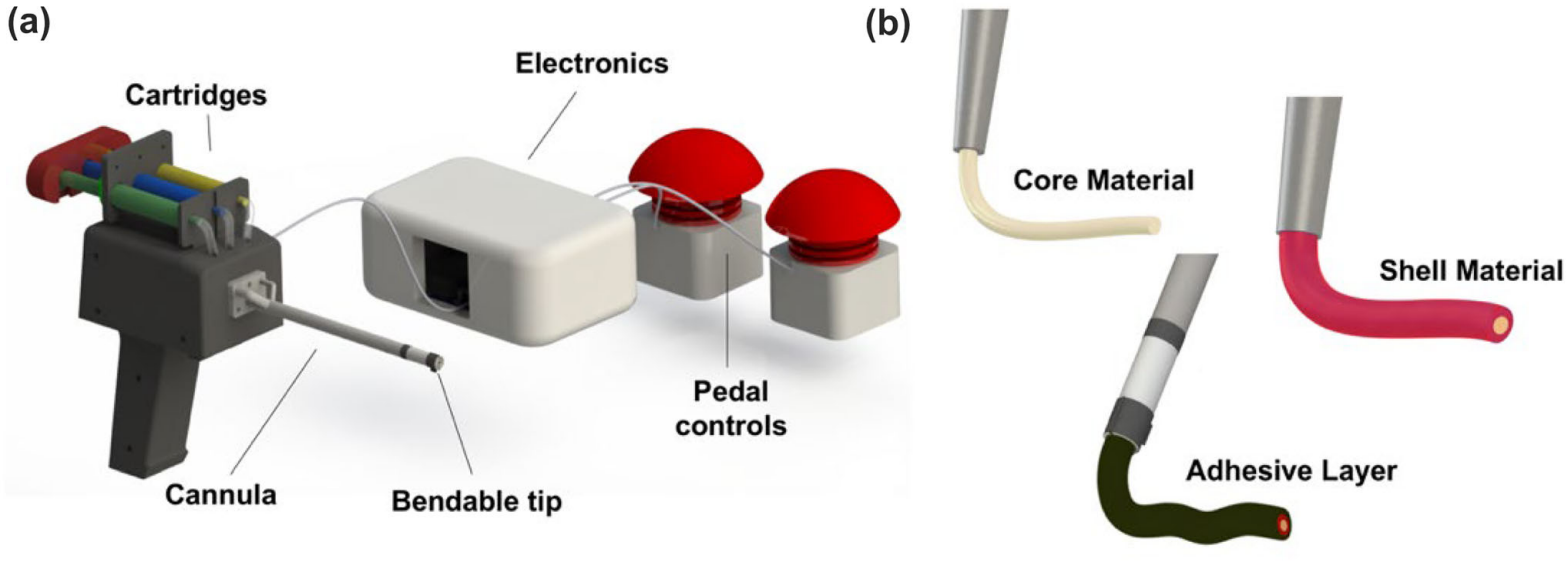
Depiction of the overall device and its components. (a) Representation of the extrusion system and its control interfaces; (b) representation of the simultaneous coaxial extrusion process for three different materials through the coaxial channels of the cannula.

As depicted in Fig. 1a, the extrusion system includes an arthroscopic cannula made of three coaxial channels ending with a flexible tip, a handheld case comprising an actuation system controlled through pedals and cartridges containing the different materials. The cannula was designed to be interfaced to the case (on one side) and the bendable tip (on the other side). The possibility to bend the tip once the instrument is inserted within the arthroscopic cavity allows modulating the orientation of the material extrusion, reaching up also cartilaginous lesions usually not achieved by conventional arthroscopic tools. The tip bending is controlled by the motorized cable actuation system, which is inside the case and driven by user-controlled foot pedals: one pedal serves to bend the tip, while the other one releases it. The case is designed to include the motorized actuation system and to host the cartridges. The cartridges are connected to the cannula inlets through three silicon polymeric tubes. On the backside of the syringes, a 3D printed component allows applying controlled load to the three cartridges simultaneously. The channel sections are developed with the aim to obtain an extrusion with a controlled volume: this feature allows a uniform deposition of the coaxial-shaped materials with uniform velocity.

The three-channels-based cannula permits the simultaneous extrusion of three different substances. The central channel (named core) delivers the therapeutic element (*e.g.,* a cell-laden biomaterial). The core channel is surrounded by two outer channels, named shell and primer, respectively. The material flowing through the shell is thought to protect the cells embedded in the core material, while the primer should ensure the adhesion of the overall extruded structure to the targeted surface, acting as surgical glue. The design of the cannula and its channels would allow the concentrical extrusion *in-situ* of the three different materials, as shown in Fig. 1b.

### Design and Fabrication of the Device

#### Design of the Cannula

**Figure 2:**
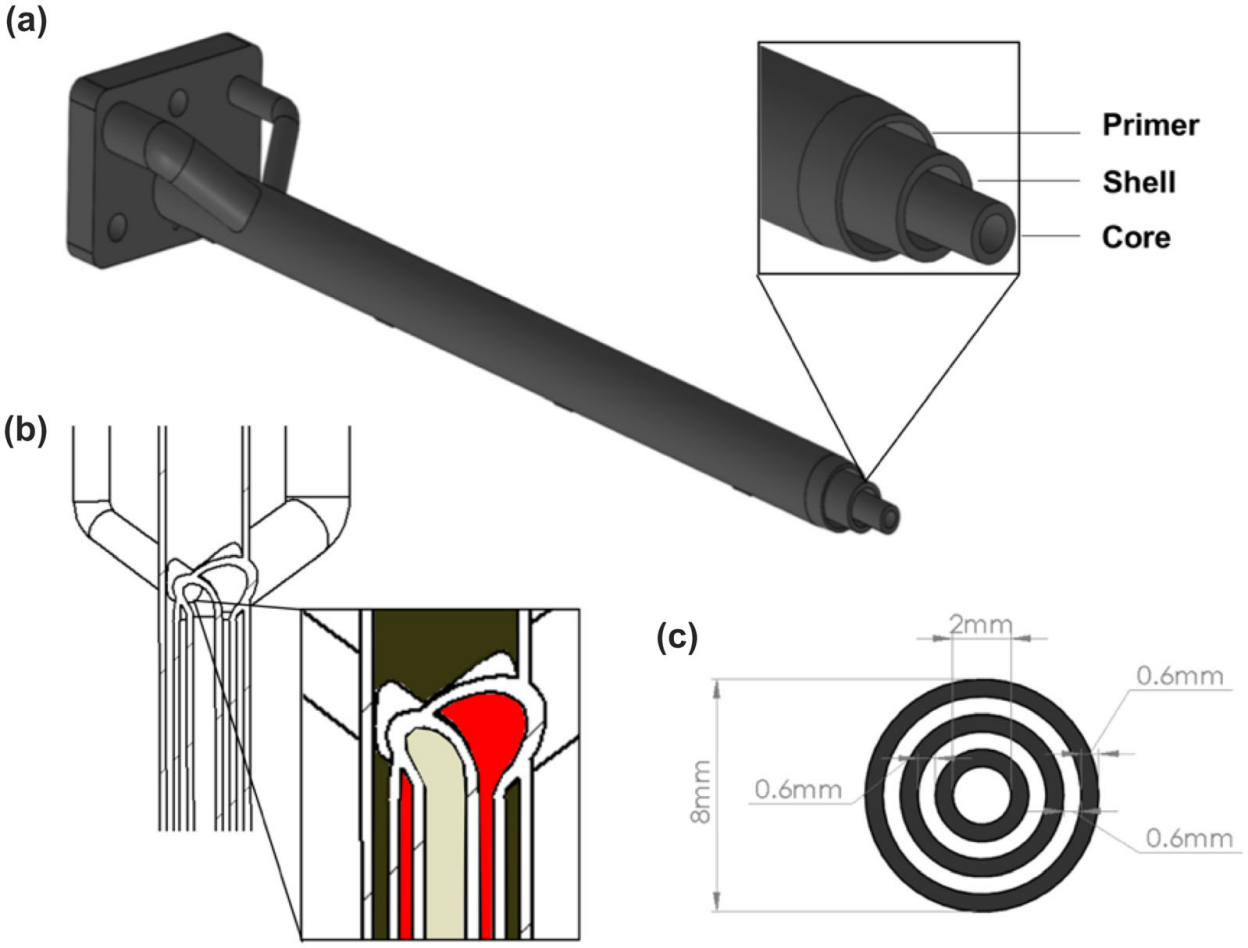
Design of the cannula: CAD of the three coaxial channels with progressive increase of the length from the primer to the core (*step* configuration); (b) convergence chamber with colored areas highlighting the lumens for the biomaterials flow (beige: core; red: shell; petrol: primer); (c) cross-section and dimensions of the channels.

As mentioned, the design of the cannula allows printing different materials simultaneously, thanks to three concentric channels, named core, shell, and primer (Fig. 2a). Silicon polymer tubes (RS PRO Silicone Transparent Silicone Tubing, code: 667–8448, inner diameter: 3 mm, outer diameter: 4.8 mm) connect the syringes (volume: 5 mL each) to the cannula channels. The distal flange of the cannula is fixed to the case by screws. The three channels unite into a convergence chamber where they assume a coaxial configuration, as sketched in Fig. 2b. The three concentric channels allow the materials to flow separately; the proximal end of the cannula has a *step* configuration due to the progressive increase of the channels’ length, from the primer to the core (see detail in Fig. 2a). This feature permits an easy interface with the bendable tip, as explained in the following sections. The cannula, similarly to arthroscopic tools already described in the literature,^4,11,17,37^ is 140 mm long with a total external diameter of 8 mm and was fabricated through direct melting laser sintering technology (DMLS), using AISI 316L stainless steel.

The core diameter is 2 mm. This dimension guarantees the extrusion of a considerable volume of material, without excessive shear stress values. The annular sections for the shell and the primer materials have a radius of 600 µm. The thickness of the walls is the smallest allowed by the manufacturing technology (*i.e.,* 600 µm) to avoid clogging between each layer. A scheme of the cannula cross-section is shown in Fig. 2c.

An attempt was made, in which an annular section of 450 µm was pursued, to minimize the overall device diameter. However, with these dimensions, multiple occlusions occurred (Supplementary Material, Figure S1), making the device not usable and suggesting that the above-mentioned dimensions are the minimal ones for implementing this concept.

#### Design of the Bendable Tip

**Figure 3:**
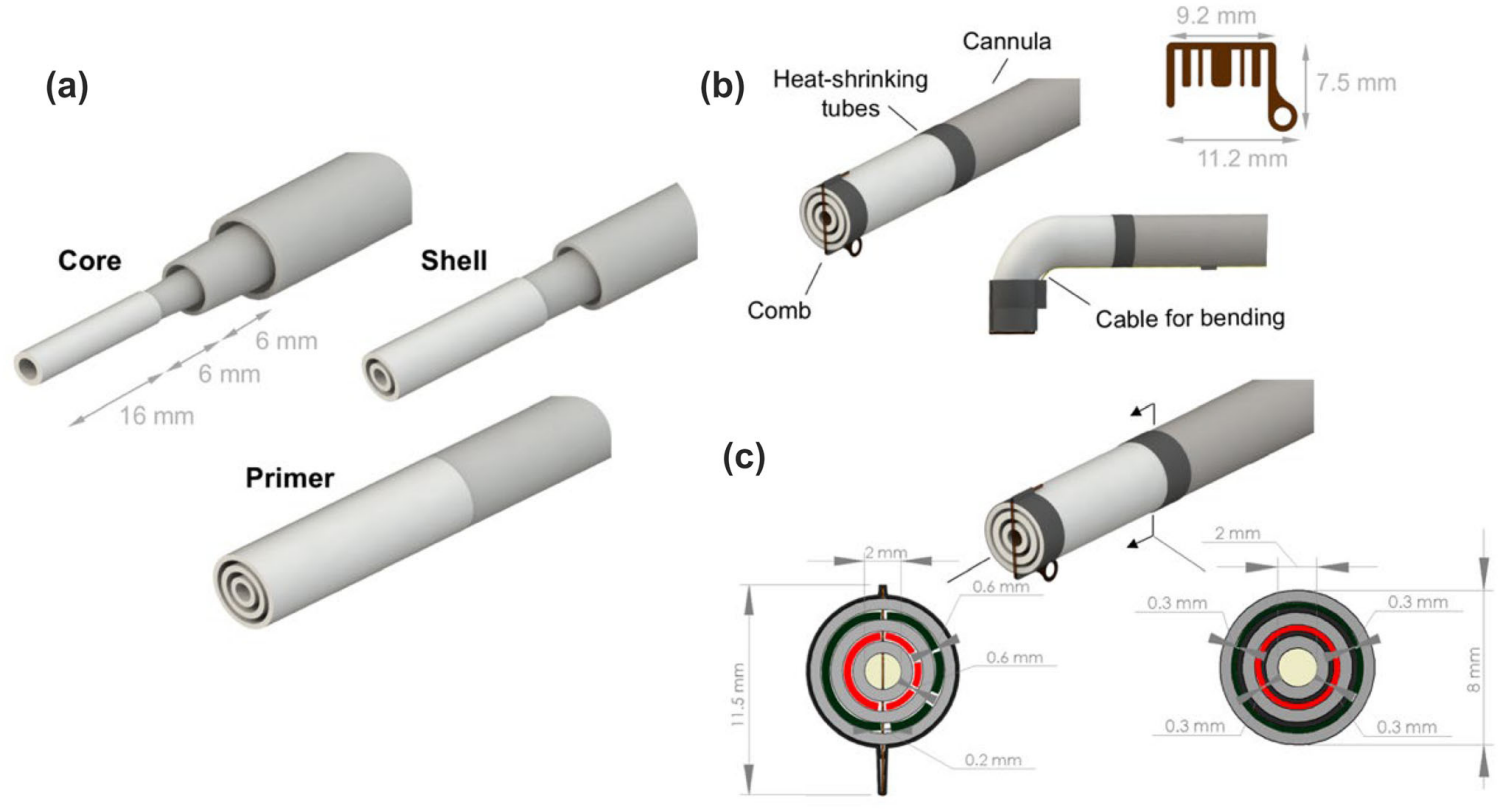
Design of the bendable tip: (a) length of the single bendable elements; (b) bendable tip components including the metal comb and the heat-shrinkable tubes; (c) lumen restriction due to the presence of the heat-shrinkable tubes. Biomaterials are represented by colored layers.

Similar to the cannula, the flexible tip is organized in three different coaxial channels. These are designed to be assembled with the cannula and to be bent when desired. The length of the flexible tip is optimized, taking into account the radial distance from the arthroscopic axis and the expected bending angle. According to,^17^ the maximum distance allowed from the arthroscopic axis is 10 mm, while the bendable angle should range from 0 and π/2: at 90° bending, the tip thus behaves as an arc of a circle of ~ 16 mm. The initial length of the flexible tip inner channel was set to 16 mm while the length of the outer channels was optimized consequently (Fig. 3a). The overall length of the shell and the primer channels is 22 and 28 mm, respectively. The diameters are the same as the cannula channels.

To guarantee the coaxiality between the three channels of the flexible tip, a comb-shaped metal foil was manufactured in AISI 316 steel alloy using electrical discharge machining (AP 200 L, Sodick, Japan). It is thought to be inserted at the proximal extremity of the flexible tip. This component is designed to keep the structure stable during bending, maintaining the section of the extrusion lumens as constant as possible. In addition, it acts as an anchoring point for the actuation cable.

The connection between the flexible and the rigid channels is achieved by using three 5 mm long heat-shrinkable tubes. Each tube allows connecting the polymeric (flexible) channel with the corresponding cannula (rigid) one. The presence of this element decreases the extrusion lumen by 300 µm. An additional heat-shrinkable tube is also used to cover the end of the tip (including the comb) to prevent the surrounding knee tissues from being damaged by its sharp edges during the intra-operative phase. The bendable tip, including all its components and the dimensions of the lumen restrictions, is shown in Figs. 3b and 3c.

## Preparation of Materials and Rheometric Analyses

A polymeric solution made of sodium alginate (SA, W201502, Sigma-Aldrich), Pluronic F127 (P2443, Sigma-Aldrich), and human chondrocytes (cat.# 402-05a, Cell Application, Inc.) was employed in the core channel. Human chondrocytes were thawed and expanded in culture with Growth Medium (cat.# 411–500, Cell Application, Inc). The intermediate shell layer was made by only Pluronic F127. Pluronic F127 doped with cellulose nanofibers (CNFs, Valida Visco-S, fibrillated cellulose in water, solid content: 3.0 ± 0.2% wt.) was investigated for the primer channel as adhesive layer. Previous evidence suggested that the combination of SA and Pluronic was suitable for hosting cells and promoting tissue regeneration,^7,35^ while the encapsulation of CNFs into hydrogels improved the adhesion strength onto the cartilage.^28,48^

The SA-Pluronic solution (2% w/v SA, 20% w/v Pluronic F127) was prepared in deionized water. The SA was firstly dissolved at 60 °C while stirring for 2 h; then, the solution was placed in an ice bath while the Pluronic F127 was gradually added and stirred for 1 h. Once dissolved, the solution was kept in the refrigerator at + 4 °C until use, and cell encapsulation (density: 10^6^ cells/mL) was performed maintaining the solution cold with ice.

The Pluronic F127 was also used for the shell conduct. Briefly, the Pluronic (20% w/v Pluronic F127) was gradually added to deionized water and stirred in an ice bath for 1 h and then the solution was stored in the refrigerator at + 4 °C until use. A red tissue marking dye (Trajan, Davidson Marking System) was added to the Pluronic solution to visually discriminate it from the core and primer formulations for the material extrusion verification.

The primer conduct was filled with a Pluronic-CNFs solution (20% w/v Pluronic F127, 0.5% w/v CNFs) to improve the adhesion strength of the printed construct to the surrounding tissue, as suggested by the state-of-the-art.^28,49^ CNFs were added to the Pluronic solution in an ice bath while stirring for 1 h. Once dissolved, the solution was kept in the refrigerator at + 4 °C until use A black tissue marking dye (Trajan, Davidson Marking System) was added to the SA-Pluronic solution to visually discriminate it from the shell and primer formulations for the material extrusion verification.

A rheometer (MCR 302, Anton Paar GmbH, Ostfildern, Germany) equipped with an H-PTD 200 temperature control device was used with a plate-plate geometry (diameter: 25 mm) to perform rheological measurements for the analysis of the material viscosities. The temperature was set at 25 °C, and all materials were left 5 min between plates to achieve the equilibrium temperature. The viscosity against the shear rate (range: from 0.1 to 1000 s^−1^) was investigated to model the viscosity of the material according to the following power law:^14^

1 $$\upeta =\mathrm{K}{\dot{\upgamma }}^{\mathrm{n}-1}$$

where η is the dynamic viscosity, $\dot{\upgamma }$ is the fluid shear rate, and K and n are the consistency index and the flow behavior index, respectively. Through linear interpolation of the shear rate sweep test curve, K and n were determined and used to model the viscosity of the fluids for the computational analyses.

Moreover, temperature ramp tests were performed for all materials varying the temperature from 4 to 40 °C and keeping a constant shear rate at 50 s^−1^.

## Computational Fluid-Dynamic Analyses

Computational fluid-dynamic (CFD) simulations were carried out to support the device dimensioning and to evaluate the flow-related parameters. The flows within the tree channels were simulated separately, and the above-mentioned non-newtonian fluids were implemented. The fluids were modeled according to the power-law equation reported in Eq. (1), whose coefficients came from rheological measurements (see “Preparation of Materials and Rheometric Analyses” section). The solution made of SA, Pluronic F127, and human chondrocytes was considered for the core channel, the Pluronic F127 only was considered for the shell, while the Pluronic F127 with CNFs solution was considered for the outer channel. The average element size was set at ~ ${10}^{-5}$ m (~ 7e^6^ tetragonal elements) and the convergence criterion was set at ${10}^{-5}$. The laminar flow module within COMSOL Multiphysics 6.0 was used to mesh the fluid volume and to solve the steady-state Navier–Stokes equations. A geometrical symmetry along the flow direction has been exploited to facilitate the computational effort of the core and the shell channels. An average pressure equal to zero was imposed on the outlet section, and the no-slip condition was set on the channels’ walls. As inputs for the computational model, different plunger velocities (0.016, 0.16, and 1.6 mm/s) were considered and the fluid domain ranged from the syringe to the straight tip. These velocities came from the standards publication (BS EN ISO 7886–1:2018) followed for the extrusion characterization (see Characterization of Coaxial Printing”). The machine used to run the CFD simulations held 32 GB of RAM and 8 cores. In Supplementary Material, Figure S2 shows the components considered in the simulation environment for the core channel and its relative boundary conditions.

### Bendable Tip Fabrication and Actuation Module

#### Fabrication of the Flexible Elements

A dip-coating approach was used for manufacturing the flexible tip.^13^ Customized stainless-steel springs were used as the skeleton and the internal matrix of the different channels. The thickness of the spring filament was equal to 400 µm; the internal diameters were chosen to fit adequately with the cannula channels and were supported by the simulation results (see “Rheometric Tests and Computational Simulations” section). During the fabrication procedure, the spring was inserted around a cylindrical metal holder and then immersed for a few seconds in a transparent and pre-vulcanized commercial latex solution (Prochima EM501K1 Latex).^36^ The thickness of the latex wall was determined by the immersion cycles. This was driven by the Landau-Levich equation for a Newtonian fluid:^13^

2 $$\mathrm{h}=\frac{0.94({\mathrm{\eta v})}^\frac{2}{3}}{{\upgamma }^\frac{1}{6}{(\mathrm{\rho g})}^\frac{1}{2}}$$

where $\eta$[Pa s] is the dynamic viscosity of the fluid, *v* [mm/s] is the extraction velocity, $\gamma$ [N/m] is the fluid superficial tension, $\rho$ [gr/cm^3^] is the density and *g* [m/s^2^] is the gravity. Considering the latex as the fluid, and taking into account a constant extraction velocity equal to 10 mm/s, the thickness of the latex wall per one immersion cycle was equal to 200 µm. Springs and supports were removed from the latex bath at 10 mm/s to avoid any inhomogeneity and, after one hour of drying at room temperature, the spring covered with a thin layer of latex was extracted and ready to be interfaced with the channels. The main phases of this process are depicted in Supplementary Material, Figure S3.

At the end of the process, 5 mm long heat-shrinkable tubes made of polyolefin were used to link the flexible components to the rigid channels: these tubes underwent a few seconds of heating at 200 °C to shrink them, thus fixing both parts together.

Using the springs for fabricating the flexible channels prevented the risk of lumen occlusion during tip bending. With equal outer diameters, this risk increases when the channel wall size decreases.^26^ The risk of lumen occlusion (also known as kinking) is described by the following Eq. (3), in which the critical radius of curvature of the channel after which the kinking occurs is calculated as:

3 $${\mathrm{R}}_{\mathrm{c}}=\frac{\left(1-{\nu }^{2}\right){\mathrm{r}}^{2}}{k\mathrm{h}}$$

where r is the outer radius of the duct, h is the wall thickness, ν is the Poisson's modulus and *k* is a constant between 0.72 and 1.14.^34^ Taking into account the dimensions specified in “Design of the Bendable Tip” section, the results of Eq. (3) lead to the necessity to use the dip-coating approach. To furtherly demonstrate the validity of these results, a second strategy based on molding (thus to obtain simple polymeric tubes, without springs embedded) was also attempted and compared with the first one.

#### Characterization and Testing of the Bendable Tip and Actuation Module

The force required to bend the final coaxial tip as a function of the bending angle was measured through an Instron testing machine (model 2444, Instron, Norwood, MA, USA). The results were used to extract suitable specifications for the cable-based actuation system. To perform this test, the cannula including the bendable tip was clamped to the machine while the actuation cable was connected to the load cell (F_max_: 10 N) to record the bending force required by the tip to be rotated up to 90°.

The results of this test, presented in Sect. 3.2, allowed a proper design of the cable-based actuation system, consisting of a motor and a transmission system.

A DC micromotor (DC-Gearmotor, Faulhaber 1512U003SR 324:1) equipped with a built-in optical two-channel encoder and maximum torque of 30 mN·m, was chosen for the actuation. The transmission components consisted of a 3D-printed part (ProJet MJP3600, 3D systems, USA, material: VisiJet M3 crystal) connected to an M6 threaded screw. The rotation of the screw due to the motor allows the linear sliding of the 3D printed part to which the actuation cable is connected. The pulling of the cable, linked to the comb, allows the bending of the tip. The entire actuation module was housed and fixed inside the device handheld case. Based on the direction of the motor rotation, the cable is pulled (and thus the tip bent) or released. The direction and the starting-up of the motor are managed by two different pedals. The motor is connected to an Arduino microcontroller, and its parameters are controlled through the Matlab software. All these components were assembled and tested, and the capability of the complete device to rotate the tip was assessed.

### Characterization of the Extrusion System: Coaxial Printing

#### Material Extrusion Test

An extrusion test was carried out to assess the pressure required by the device to coaxially extrude the biomaterials. This test aimed to confirm the bendable tip behavior and validate the chosen design dimensions. The Instron testing machine was employed to press the materials loaded in three 5 mL cartridges and to read the corresponding pressures required through a ± 1 kN load cell. The solution, including human chondrocytes, was loaded into the core syringe, while the other materials, prepared as described in “Preparation of Materials and Rheometric Analyses” section, were loaded into the shell and primer syringes. Compression was carried out at a speed of 1.6 mm/s, according to the standard velocity defined in BS EN ISO 7886–1: 2018, for a total displacement of 2 mm. This displacement corresponded to 0.13 mL of extruded material per syringe. The test was performed holding the tip straight and bent, in order to evaluate the influence of bending on the extrusion parameters and printability. The test was performed keeping the material at a temperature of 25 °C.

#### Cells Viability After Extrusion

Cells at a density of 10^6^ cell/mL were gently encapsulated into the core channel formulation, composed by Pluronic and SA, at 4 °C before starting the tests, and control was cast into a petri dish for comparison. Then, the cell-laden solution was moved into a 5 mL cartridge and extruded using the triaxial cannula in the straight and bent configuration. After extrusion, the core was crosslinked for 10 min by adding on the top a solution of calcium chloride (CaCl_2_, Merck) at a concentration of 1 mM. Then, the Growth Medium was added, and the crosslinked cell-laden hydrogels were placed in an incubator at 37 °C and 5% CO_2_. Cell viability was determined after 24 h with the Live/Dead assay (Thermo Fisher Scientific, USA). The hydrogels were washed thrice with phosphate buffered saline (PBS) and then incubated with ethidium homodimer-1 (4 μM) and calcein-AM (2 μM) for 45 min at 37 °C according to the manufacturer’s instructions. After three washing steps with PBS, cell viability was evaluated by a confocal microscope (C2s system, Nikon, Japan) equipped with fluorescein isothiocyanate and TRITC filters to assess viable (green) and dead/injured (red) cells. Four 2D planes were captured throughout the construct section in height.

### Ex Vivo Test on a Human Cadaver

A test on a human cadaveric knee was performed to furtherly validate the bending and extrusion procedures of the device in an arthroscopic environment. The cadaver test was performed on a male knee available at a dedicated teaching and research surgical center (iClo s.r.l., Verona, Italy), fully accredited for cadaver management. All procedures were carried out in a fully equipped surgical room, with proper individual protection devices and clothes. The left knee belonged to a person with the following features: age: 75; gender: male; race: Caucasian. The test followed the antero-medial and antero-lateral approaches. For this purpose, two cartilaginous defects of approximately 10 mm were made on the loading surface of the medial femoral condyle and the lateral femoral condyle, respectively. These lesions were created to recall as much as possible the typical cartilage issues found in human knees: the first lesion was larger, with variable depth and rough margins, representing the typical consequence of the arthrosis disease; the second lesion was more circular and with sharp margins, more similar to cartilaginous tissue trauma. In particular, this second induced lesion was made in a position hard to reach by the traditional surgical tools. The cannula and the flexible tip were inserted inside the knee as a normal arthroscope from a small hole created ad hoc on the external skin.

## Results

### Rheometric Tests and Computational Simulations

Representative curves of the rheometric measurements are shown in Supplementary Material, Figure S4. From these curves, K and n values were extracted for all materials, as reported in Table 1.

**Table 1:** K and n values for the core, the shell and the primer materials, fitted according to Eq. (1), obtained by the shear rate sweep curves.

	Material	K [Pa s^n^]	n
Core	Pluronic F127 + SA + Cells	6.21 ± 2.44	0.42 ± 0.08
Shell	Pluronic F127	0.57 ± 0.22	0.22 ± 0.11
Primer	Pluronic F127 + CNFs	0.73 ± 0.08	0.40 ± 0.01

Results are expressed in terms of mean ± standard deviation, n = 5

The addition of cells and SA to Pluronic F127 considerably increased the consistency index of the material (from an average value of 0.57 to 6.21), as well as the n value, while the presence of the CNFs only significantly altered the n value. The results found are in line with the ones already reported in the scientific literature.^10,27^ In Supplementary Material, Figure S4, also shows how the viscosity of each formulation is dependent on the temperature, with a trend that differs depending on the material composition. Shrinky et al. reported a similar trend of Pluronic.^46^

**Figure 4:**
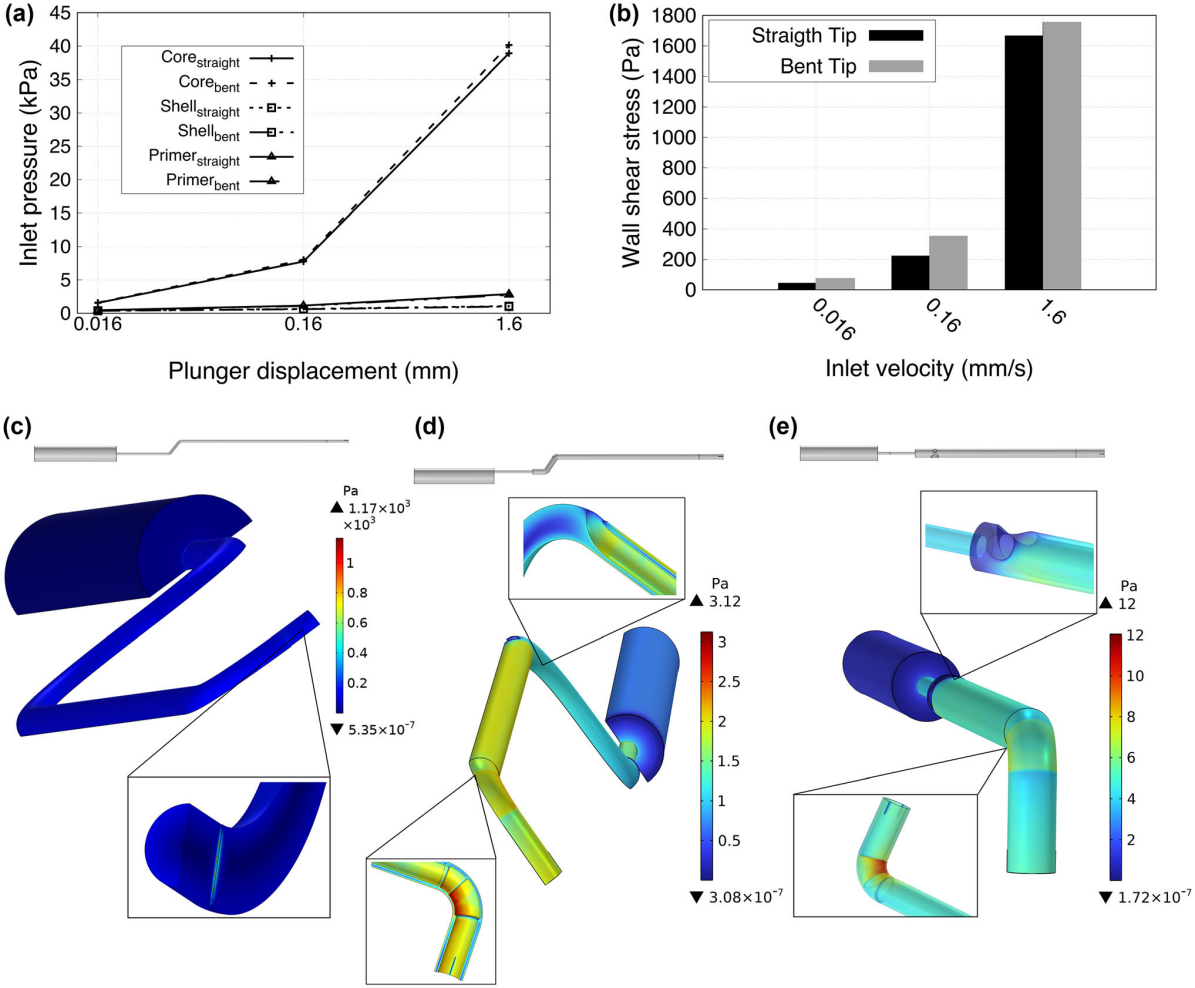
CFD simulation results: pressure required for extrusion different biomaterials in different configurations of the tip (a) and the maximum shear stress values of the core channel in straight and bent condition (b), for different velocities imposed at the inlet. 3D contour plot of the estimated shear stress over the different channels (and their relative CAD representations), for the core (c), shell (d), and primer (e), when 1.6 mm/s of velocity is imposed at the inlet.

The flow parameters were mainly influenced by the different material properties and different geometrical layouts of the three channels. Figure 4 resumes the results of the CFD simulations.

In Fig. 4a the required pressure to extrude the different biomaterials through the channels is reported, while Fig. 4b shows the maximum shear stress values obtained in the core channel, varying the extrusion velocity from 0.016 mm/s to 1.6 mm/s. The extrusion pressure obtained for the core is sensibly higher with respect to the other channels (~ 1 order of magnitude greater). This is due to the higher dynamic viscosity of the core solution with respect to the shell and primer ones, as reported in Section Table 1. The core channel was especially considered for the estimation of the wall shear stress due to the presence of chondrocytes within the fluid. The maximum values found for this parameter are presented in Fig. 4b, and are well below the threshold for guaranteeing high cell viability, which is ~ 5 kPa.^6^ As the same figure shows, the tip bending does not affect the shear stress significantly. The element affecting more the shear stress is instead the presence of the metal comb: this component in fact produced a ‘flow cut’, which considerably increase the shear stress (although keeping it within a safe range) as shown in Fig. 4c. The bendable tip resulted the most delicate point regarding the shear stress also for the other channels, due to the progressive restriction of the cross-section caused by the presence of the heat-shrinkable tubes. This is reported in Figs. 4d and 4e, where the shear stress, over the shell and the primer channels, is presented when 1.6 mm/s of velocity is imposed at the inlet. Overall, the tip and the convergence chamber are the most delicate areas for the inception of shear stresses. Although the required pressure is different for the three channels, it is possible to obtain a volume-controlled and coaxial extrusion, due to a modification of the core and shell channels. In particular, by narrowing the interface sections between the cartridges and these channels (of a quantity equal to the ratio between the outlet cross-sections), it is possible to control the reduction of the flow rate, thus making equal the outlet velocities from the three channels (Supplementary Material, Figure S5). In this way the extruded volume can be controlled and the coaxial structure of the printed materials is guaranteed.

### Fabrication and Characterization of the Bendable Tip

**Figure 5:**
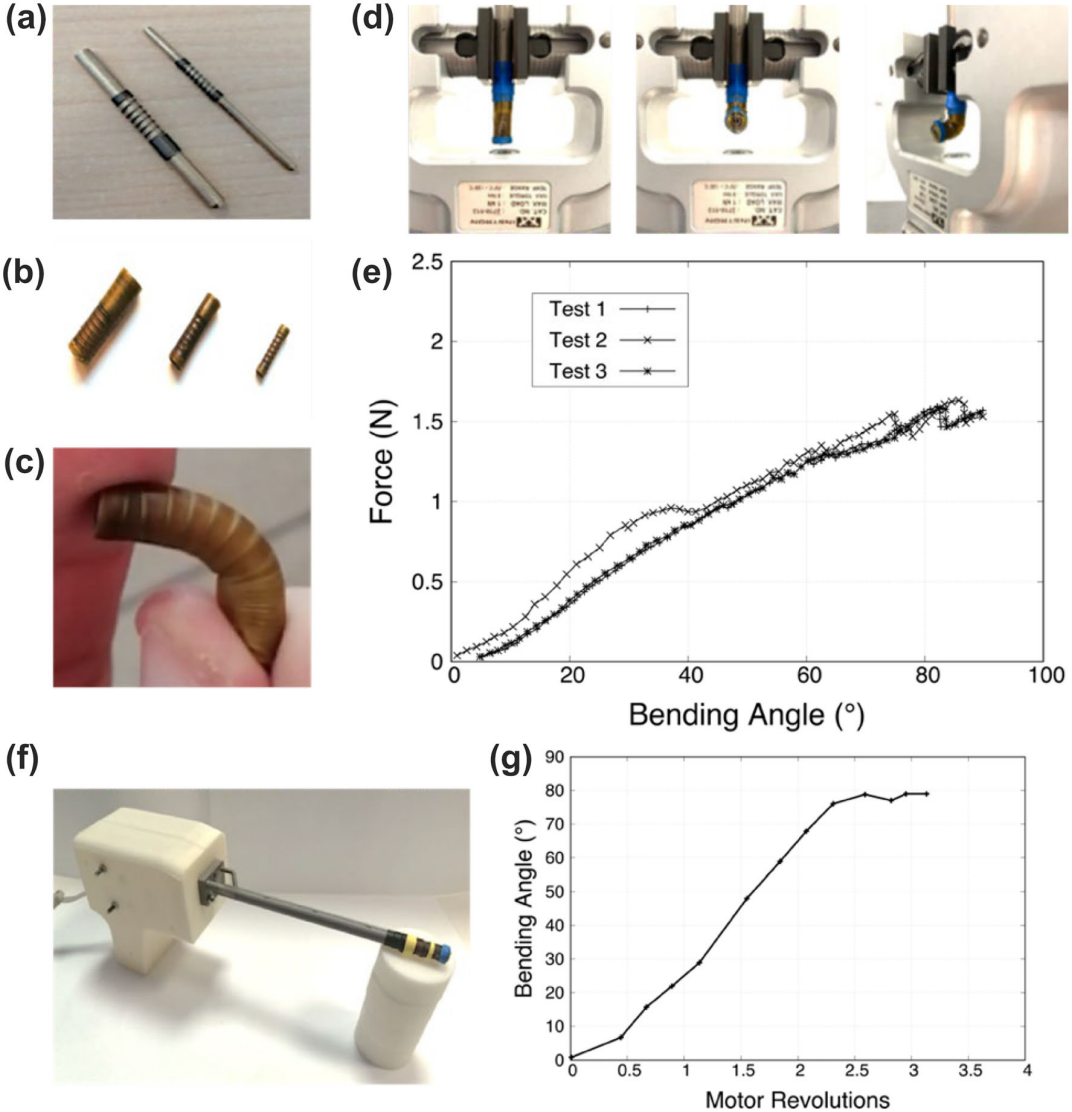
Fabrication and characterization of the tip bending behavior: springs arranged in the metal holders ready to start the dip-coating approach (a): three bendable tips made of springs-embedded latex (b); demonstration that the tip can be easily bent without kinking phenomena (c); experimental set-up at the Instron machine used to measure the force needed to bend the tip at different angles (d); results obtained in terms of traction force needed *vs*. bending angle. Three experiment was carried out in triplicate (e). Assembled device with a case containing the motor (f); graph reporting the actual bending angle of the tip with respect to the motor revolutions (g).

The fabrication process of the flexible parts through the dip-coating method, as described in “Fabrication of the Flexible Elements” section, led to channels with the desired wall thickness (Figs. 5a and 5b), namely ~ 600 µm for each duct, including the spring filament (having a nominal diameter of 400 µm) and ~ 100 µm of latex coating. These dimensions allowed the fluid to flow continuously from the rigid cannula to the flexible tip.

The presence of the internal spring in each hollow component assumed the relevant role of enhancing the radial stiffness of the tubular structures, thus reducing the possibility to undergo kinking during flexion (Fig. 5c) and allowing at the same time an elastic return to the starting position after bending (Supplementary Material, Video 1). The shape of the tip is preserved by the hyperelastic nature of the latex, which is not prone to plastic deformations. The critical radii of curvatures obtained using Eq. (3) were 3.77, 13.03, and 30 mm for the core, shell and primer, respectively. These radii were incompatible with the bending curvature targeted by the tip (90°), thus leading to kinking phenomena along the outer channels, if traditional channel architectures and materials were used. This is confirmed by the results shown in Supplementary Material, Figure S6: here, the lumen occlusion is evident in latex tubes fabricated by simple molding. The spring-based dip-coating method, instead, allowed overcoming this issue. As described in “Fabrication of the Flexible Elements” section, each flexible channel was then fixed to the rigid cannula through 5 mm-long heat-shrinkable tubes. Representative pictures of these passages are reported in Supplementary Material, Figure S7.

The results of the assembled tip bending characterization are shown in Fig. 5d, and 5e. Figure 5d shows photos of the experimental set-up composed of the cannula clamped vertically in the tensile test machine with the tip straight (left) and with the tip bent at 90° (center, right). The test aimed to measure the force required to bend the tip from 0° to 90°. Figure 5e shows that the tip can be bent up to 90°, exerting an average force equal to 1.5 N with an almost linear trend over the bending angle.

The force values obtained from this test were used for dimensioning the actuation system. This system was integrated inside the device to actuate the tip, using the components described in “Characterization and Testing of the Bendable Tip and Actuation Module” section. The device, enclosed in a case that hosted the actuation mechanism, is shown in Fig. 5f. The tip bending was then activated through the motorized system and the actual bending angle was measured and plotted against the motor revolutions read by an encoder. The results are shown in Fig. 5g.

### Characterization of Coaxial Printing

**Figure 6:**
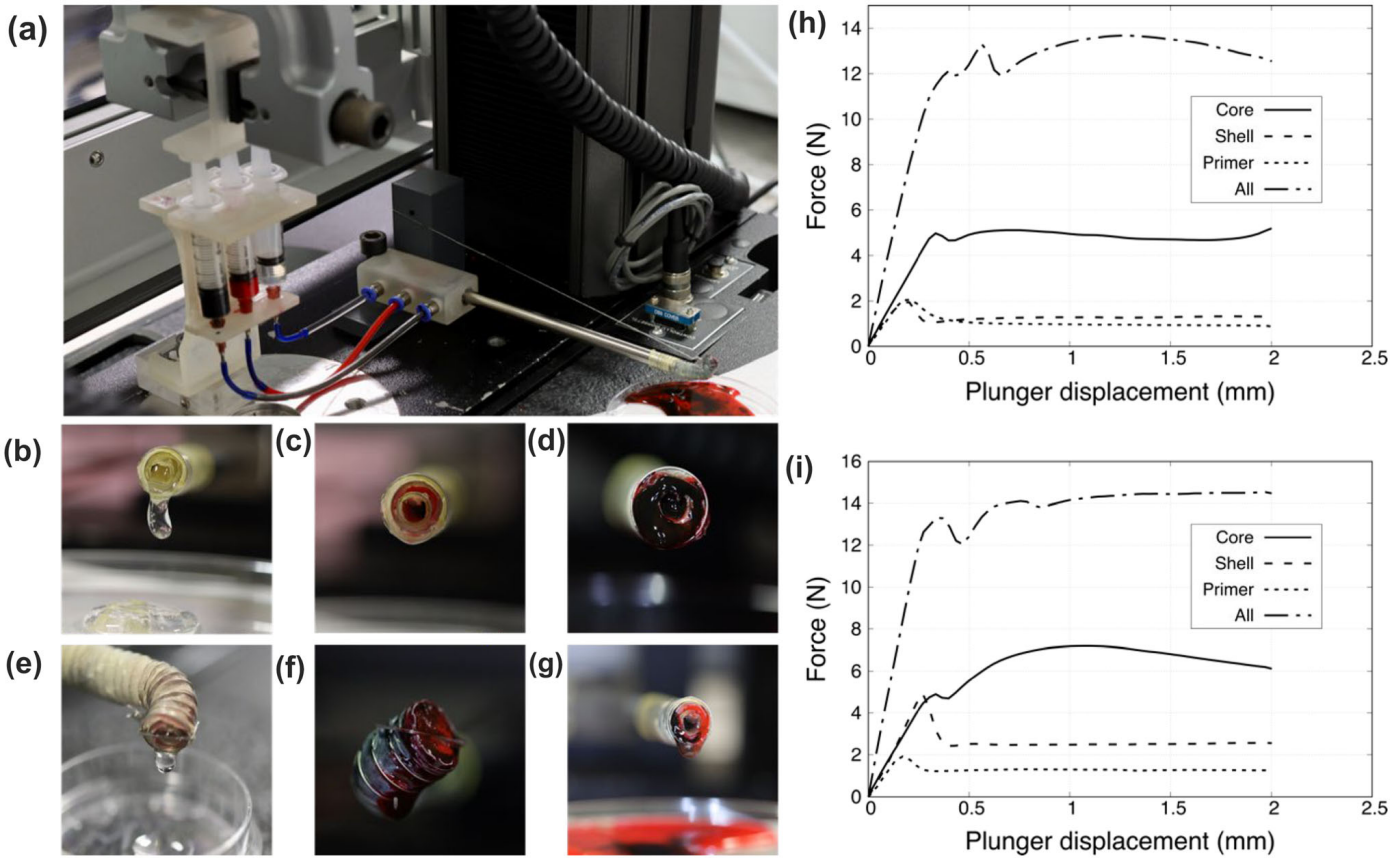
Extrusion characterization: set-up including the device placed at the Instron testing machine (a); one by one biomaterials extrusion in straight condition firstly (core in beige (b), shell in red (c), primer in petrol (d)), and in bent condition (e, f). Simultaneous extrusion with the tip straight (g). Quantitative force measurement: single and simultaneous extrusion force measured in straight condition (h), single and simultaneous extrusion force measured in bent condition (i).

Extrusion tests were performed to qualitatively assess the ability of the device to guarantee material deposition and a proper geometric arrangement in both straight and bent conditions. Pictures showing the experimental set-up and the extrusion of the materials are shown in Fig. 6. This test was also performed by interfacing the extrusion system with the Instron machine, to measure the force required to extrude the materials. The set-up used is shown in Fig. 6a, where are visible the device (including the cannula and the bendable tip), the cartridges containing the biomaterials and the Instron 1 kN load cell. The materials were firstly extruded one by one while keeping the tip straight, as represented by Figs. 6b (core extrusion), 6c (shell extrusion), and 6d (primer extrusion). Then, the same test was repeated keeping the tip bent (Figs. 6e and 6f). Finally, the materials were extruded together simultaneously, in order to analyze the force required (Fig. 6g). The values of force required to extrude are plotted in Figs. 6h and 6i.

As it concerns the single channels, the highest values were recorded for the core extrusions, both in straight condition (~ 5 N) and in bent condition (~ 7 N). The bending of the tip does increase slightly the extrusion force. A less viscous composition of the materials flowing inside the shell and primer, resulted in a significant reduction of the extrusion force (between 1.5 and 2.5 N). Slightly larger forces were observed in the case of simultaneous extrusion: to deliver 0.4 mL of substances, a force of ~ 14 N was needed for the straight condition, while less than 16 N were required for the bent condition. The device was able to extrude the biomaterials considered without any discontinuity, in both straight and bent conditions, and very low values of force were required. Finally, a small quantity of material was extruded and analyzed through a Hirox 3D digital microscope. Fig. S8 shows the coaxiality of the construct and its thickness. The diameter of the printed structure matches the cannula dimensions (~ 8 mm); the thickness is about 2.5 mm.

### Cell Viability Analysis

**Figure 7:**
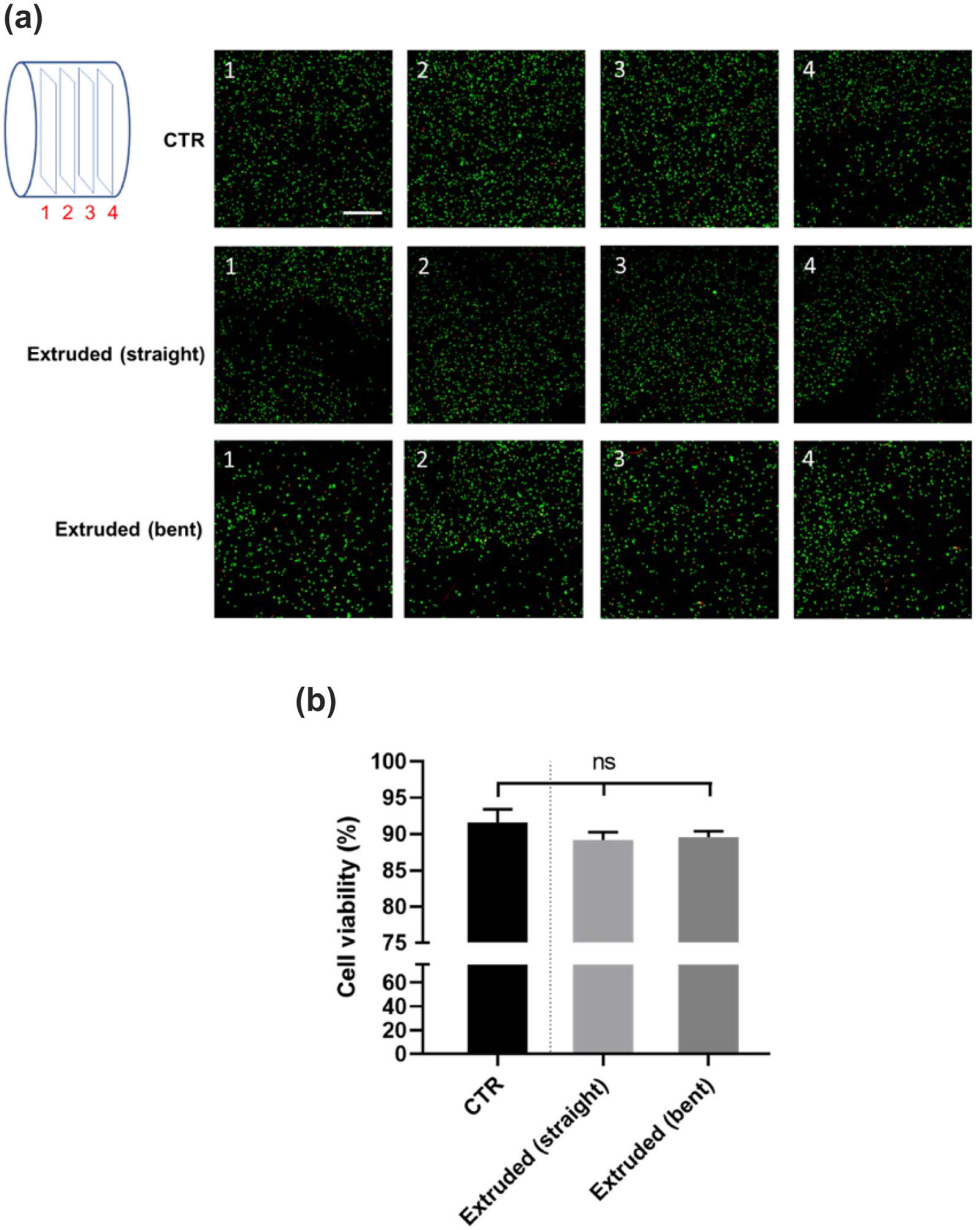
Confocal images (4x) of the cell-laden core hydrogel after using the Live/Dead assay, 24 h on the cast (CTR) and extruded (straight and bent) formulations (a). Four planes, spaced by 250 µm, were captured to visualize the cell viability and distribution throughout the crosslinked hydrogel (green = viable cells, red = dead cells, scale bar = 200 μm). Quantitative analysis of cell viability, obtained by counting the number of viable cells over the total cell number (b).

As shown in Fig. 7, the Live/Dead assay showed that the cell-laden hydrogels extruded in both straight and bent tip configurations guaranteed a large amount of viable cells after 24 h. No significant differences in terms of cell viability were found between the extruded materials and a control (CTR) sample type, in which cell-laden materials were cast, thus without being subjected to any shear stress. The overall cell viability resulted around 90%. Similar cell distributions were also found, while comparing the three cases. Data confirmed the simulation predictions, meaning that the shear stress applied during the extrusion did not significantly affect cell viability, either in the straight and bent tip configurations.

### Ex Vivo Test on a Human Cadaver

**Figure 8:**
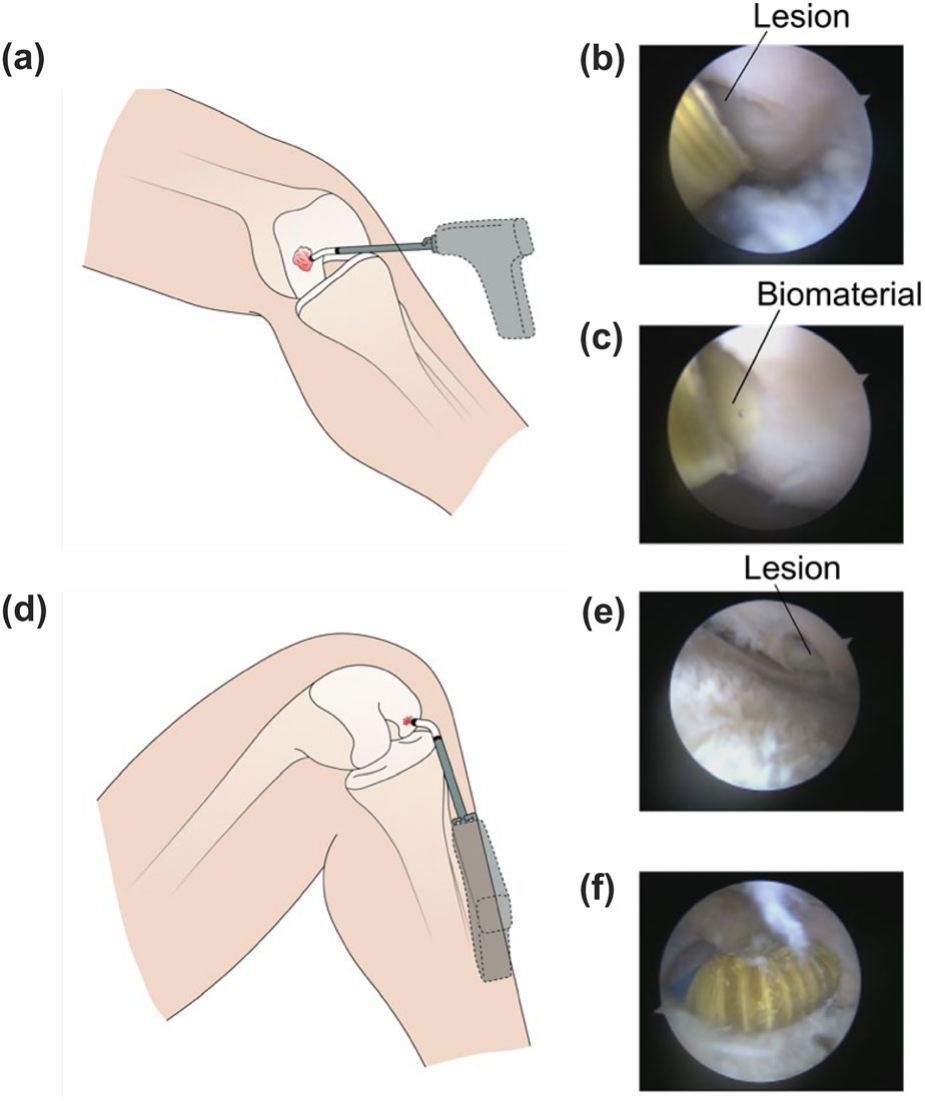
Results of the ex vivo test performed on a human cadaveric knee: (a) schematic representation of the location of an antero-medial lesion, relatively easy to reach; (b) intra-operative photo of the lesion; (c) biomaterial extrusion by the device, used to fill the lesion; (d) schematic representation of the location of an antero-lateral lesion, relatively hard to reach; (e) intra-operative photo of the lesion; (f) flexible tip bent at 90° to reach the lesion and to deliver the biomaterial there ensuring an orthogonal position of the tip with respect to the lesion.

As a final assessment, a test on a cadaveric knee was performed following the procedures described in “Ex Vivo Test on a Human Cadaver”. The first test involved the material extrusion from the core channel in correspondence to a spread lesion (diameter: ~ 20 mm) made by the surgeon on the surface of the medial femoral condyle, thus in a relatively accessible area depicted in Fig. 8a. Intra-operative images of this procedure are shown in Figs. 8b and 8c. In Fig. 8b, the presence of an acute lesion can be observed. Figure 8c shows the yellow-colored material extruded through the arthroscopic device, filling the lesion.

The second test aimed to demonstrate the capability of the device to reach more remote lesions thanks to the possibility of bending the tip. For this purpose, the surgeon made an acute circular lesion (diameter: ~ 10 mm) on the posterior part of the lateral femoral condyle surface, as depicted in Fig. 8d. This lesion, shown in Fig. 8e, was perfectly reached up by the tool once the tip was bent at 90°, ensuring an orthogonal position of the tip with respect to such a lesion, and thus allowing an efficient material delivery (Fig. 8f). The main moments of the test, such as the creation of the lesion and the biomaterial extrusion are collected within the Supplementary Material, Video 2 and Video 3.

## Discussion

The system presented in this paper was designed having the knee cartilage as the main target. However, it could be exploited to deliver biomaterials in other human joint cavities, since its dimensions are suitable to be also used in other frameworks. The small size of the device, above all in its terminal part, makes possible a minimally invasive approach to deliver the materials *in-situ*. Due to this feature, the surgeons could directly manage the parameters of the extruded material and could adapt them to the patient’s tissue.^15^ Apart from the small dimensions, the advantages of this device are mainly due to the flexibility of the tip: through this feature, the ability to reach regions that are hard to reach with traditional instruments is decisively increased. In the state-of-the-art, among the most interesting examples of in situ delivery of hydrogels for cartilage-related applications are the works of O’Connell and colleagues,^38^ and Di Bella and colleagues.^15^ However, the devices proposed in these papers require an open surgery approach and do not include a bendable tip. The arthroscopic device proposed in this paper and the additional features enabled by its bendable tip constitute an innovation with respect to these efforts.

The interest in printable hydrogels for regenerative applications (in particular for cartilage regeneration or substitution) is growing.^1,12,21,25,44,50,51^ In this framework, the possibility of simultaneously delivering different materials has advantages with respect to single material extrusion. As reported by Duchi et al*.*,^16^ using a material in a shell compartment coaxial with respect to a core, selected among photoresponsive formulations (*e.g.,* methacrylated gelatin), can guarantee shell hardening upon light exposure, ensuring the stability of the extruded core–shell structure. This solution allows better protection of cell differentiation and proliferation from crosslinking products and from the shear stresses generated during material deposition.^16^ On the other hand, increasing the adhesion of an extruded hydrogel to the cartilage tissue, *e.g.,* through the simultaneous extrusion of a primer, results in a more efficient tissue treatment and repair.^29^ The device proposed in this paper allows, for the first time based on the authors’ knowledge, to coaxially extrude three different substances at the same time: a core made of a hydrogel that can be provided with cells, if needed, a protective shell and a primer to guarantee adhesion to the target, also relying on the possibility to do it through a bendable tip. Indeed, we considered the core made of alginate-Pluronic F-127 to host cells, an intermediate shell layer made of only Pluronic F-127, and the primer formulation composed of Pluronic F-127 enriched with cellulose nanofibers whose scope is to increase the adhesion strength of the triaxial structure onto the targeted tissue (e.g., cartilage). With the materials used in this work (e.g., sodium alginate), ionic crosslinking would be a suitable approach for crosslinking the core. This may be implemented, for example, by injecting a saline solution containing calcium ions into the joint. Alternatively, we may directly add a low concentration of calcium ions to the core before printing,^22^ or even in the shell, which could diffuse into the core.^33^ All the solutions mentioned would be compatible with our device design. Other materials with similar viscosities could be easily applied with the here-mentioned device, as for example hyaluronic acid.^41^

This represents a novelty in the framework of the 3D portable bioprinters,^55^ and, in general, in the field of handheld devices for material extrusion.

The use of springs as the inner structural matrix to manufacture the bendable components allowed to avoid tube kinking and guaranteed an elastic return from bending. The fabrication through dip-coating was a convenient and fast strategy, already exploited for fabricating coatings over components in many fields *e.g.*, orthopedics applications or electronic components fabrication,^31,43^ and suitable to fabricate tips resistant to pressures and impermeable to fluids.^32^ However, in our work, we proposed for the first time the use of springs coated with latex and using the dip-coating method to avoid tube kinking and guarantee biomaterials extrusion. This approach may inspire the development of similar devices, in which lumen viability for extruding materials or for injecting liquids is crucial, even when the final tip of the device is bent. The validation of the above-mentioned main features (tip bending and coaxial extrusion) was obtained experimentally as described in “Fabrication and Characterization of the Bendable Tip” and “Characterization of Coaxial Printing”. The characterization tests ensured that the tip allowed a bending up to 90° and, at the same time, revealed that the forces needed to achieve such a bending were in the range achievable by small electrical motors. Although the presence of three concentrical springs inside the tip, the result of the test allowed the use of an actuation system small enough to be enclosed within the tool itself, keeping it easy to handle.

Other tests assessed the force required by the system to extrude the biomaterials, which were selected as hydrogel models (different ones could be used, but we expect that the rheometric characteristics would not vary much). These tests validated the outcomes of the computational simulations. Simulation results (in terms of pressure) were slightly smaller than those obtained experimentally (error: ~ 10%). This error was probably due to unexpected frictions along the paths, due to a non-ideal assembly procedure. However, the simulations constituted a useful tool to predict in advance the shear stresses along the different channels. The values obtained (1755.18 Pa for the core channel in bent configuration) were positive in view of possible cell-laden material extrusion, since shear stresses greater than 5 kPa could negatively affect the cell viability.^6^ This outcome was confirmed by evaluating the viability of human chondrocytes embedded in the core, which showed a high viability 24 h after extrusion in both straight and bent tip configurations. This paradigm may be extended in the future to other devices dedicated to biomaterial extrusion, to evaluate both the shear stresses produced given a certain geometry of the device and the pressure needed to extrude certain materials, to properly dimension the actuators of the system.

The final test on a human cadaver qualitatively demonstrated the effectiveness of the concept proposed and the potential of the device. During this test, the surgeon was able to reach remote lesions and effectively deliver the biomaterial in an arthroscopic environment.

The limitations of this study are: (i) the lack of an integrated motorized slide and pedal-based interface for full material extrusion control; (ii) the materials and components used for the development of this proof-of-concept were not entirely suitable for future sterilization and certification of the device. These steps would be the objective of future evolution and optimization of the device, in view of its possible clinical use.

## Supplementary Information

Below is the link to the electronic supplementary material.

Supplementary file1 (MOV 10039 KB).

Supplementary file2 (M4V 136499 KB).

Supplementary file3 (M4V 124706 KB).
